# Evaluation of long-term COVID-19

**DOI:** 10.18632/aging.203253

**Published:** 2021-06-26

**Authors:** Michael C. Kiefer, Samuil R. Umansky

**Affiliations:** 1 DiamiR Biosciences, Monmouth Junction, NJ 08852, USA

**Keywords:** COVID-19, late sequelae, organ-enriched miRNAs, plasma miRNAs, universal screening test (UST)

**COVID-19 late complications.** Coronavirus disease 2019 (COVID-19), which is caused by the severe acute respiratory syndrome coronavirus 2 (SARS-CoV-2), has the potential to become a long-term health problem due to persistent symptoms that arise following the acute viral infection. Many individuals who have COVID-19 make a full recovery and return to their baseline state of health. Others, however, have symptoms or other sequelae for weeks and months after initial SARS-CoV-2 infection. The constellation of symptoms and other effects experienced by patients who do not return to their baseline state of health after COVID-19 has been referred to by many names, including post-acute sequelae of COVID-19 (PASC), post-COVID condition or syndrome, and long or long-haul COVID.

Although COVID-19 initially affects the lungs, clinical and scientific evidence is evolving regarding the long-term effects of COVID-19, which can be wide-ranging in severity and duration and can affect multiple organ systems, with symptoms such as fatigue, dyspnea, cognitive dysfunction, anxiety, and depression often described. Longer term effects have been reported in all age groups and demographics and in people with asymptomatic, mild, or severe COVID-19 illness.

Emerging studies indicate a broad organotropism of SARS-CoV-2 beyond the lungs to other tissues, organs, and systems resulting in multiorgan dysfunction, such as renal complications, gastrointestinal dysfunctions, endocrine system disorders, thromboinflammation, neurological dysfunctions, dermatological symptoms, hematological manifestations, and myocardial dysfunction and arrhythmia. Cellular damage due to a robust innate immune response with cytokine production and a pro-coagulant state induced by SARS-Co-V-2 may contribute to these sequelae [1]. This broad effect of COVID-19 disease is consistent with the finding that SARS-CoV-2 gains entry to cells by binding to the cell surface receptor angiotensin-converting enzyme-2 (ACE-2), which is widely distributed on many cell types in human tissues.

Many recent studies have reported on the long-term effects of COVID-19 (see Nalbandian review [1]). Of note is a study that quantified the rates of organ dysfunction in individuals with COVID-19 after discharge from the hospital compared with a matched control group from the general population [2]. The research team from University College London tracked rates of hospital readmission in over 47,000 COVID-19 patients and the control group for all causes of mortality and diagnoses of respiratory, cardiovascular, metabolic, kidney, and liver diseases. Over a mean follow-up time of 140 days, nearly one-third of individuals who were discharged from the hospital after acute COVID-19 were readmitted and more than 10% died after discharge, with these events occurring at rates 4 and 8 times greater than the matched control group, respectively. Rates of respiratory disease, diabetes, and cardiovascular disease were also significantly higher in COVID-19 patients. The researchers concluded that patients discharged from the hospital after COVID-19 had increased rates of multiorgan dysfunction compared with the expected risk in the general population. Importantly, they suggested that urgent research is needed to establish the risk factors.

Another recent research article highlighted the persistent neurological and cognitive dysfunction-related sequelae associated with non-hospitalized, COVID-19-infected individuals termed “long-haulers” [3]. The main neurological symptoms were “brain fog” (81%), headache (68%), numbness/tingling (60%), dysgeusia (59%), anosmia (55%), and myalgia (55%).

The expansive global burden of SARS-CoV-2 infection suggests that the potential public health effects of post-acute COVID-19 are significant, even if a small proportion of infected persons have prolonged recovery or fail to return to their baseline state of health. Specialty clinics have been established both in the United States and worldwide to address the increasing need to care for these COVID-19 patients. At a recent NIH workshop on post-acute COVID-19, clinicians provided observations on the diverse needs of patients, which included multisystem symptoms (such as fatigue, mental health problems, and pain) and other signs and symptoms pointing to specific organ systems (such as renal, cardiac, pulmonary, and gastrointestinal). These patients will require individualized and multidisciplinary approaches to treatment [4], and clearly a simple, non-invasive test to diagnose early injury to various organs and to monitor progression and treatment of disease in these patients would be extremely useful.

**Universal Screening Test (UST).** Predictions or early detection of potential COVID sequelae would be very useful for disease and treatment monitoring and could be quite helpful if detected before discharge from the hospital. However, as described above, there are numerous potential late complications of COVID infection involving different organs, and their manifestations may vary from subject to subject. Thus, screening for early detection of various organ pathologies would be complicated and expensive. Recently, we proposed the development of a Universal Screening Test (UST) for early detection of potential pathologies of various organs and tissues [5]. The UST concept is based on analysis of cell-free microRNA (miRNA) signatures circulating in the blood plasma. miRNAs are short, non-coding regulatory molecules that have several advantages as potential biomarkers: (i) many of over 2000 known human miRNAs are specific to or highly enriched in particular organs, tissues, and cell types, and hence, changes in their plasma concentrations should reflect physiological and pathological processes in corresponding organs, tissues, or cell types; (ii) such miRNAs can be detected with high specificity and sensitivity in one multiplex molecular panel; and (iii) miRNAs have relatively high stability in circulation as compared with other RNA species.

The concentration of individual miRNAs in plasma depends on a number of factors, including levels of synthesis in different cells and secretion/excretion, cell death, degradation of cellular compartments (e.g., synapsis and neurites in neuronal cells), and structural form in extracellular space (such as exosomes, microvesicles, or complexed with proteins and lipids). In addition, plasma concentrations of miRNAs enriched in particular organs may be affected by changes in blood supply caused by aging-related processes, tumor growth, and other factors. Thus, in order for plasma miRNAs to be useful as biomarkers, these factors must be taken into consideration. To compensate for these variations, we have successfully used miRNA pairs (a ratio between two miRNAs) instead of individual miRNAs as pathology biomarkers [5–7]. Biomarker pairs in which miRNAs are highly correlated have proven to be most effective [5]. Our studies demonstrated that three to four miRNA pairs can be combined into an miRNA classifier to increase the overall sensitivity. The feasibility of disease detection using the UST approach has been tested in pathologies of several organ systems based on the analysis of circulating organ-enriched miRNAs: (a) neurodegeneration in different brain regions due to Alzheimer’s disease, Parkinson’s disease, frontotemporal dementia, and amyotrophic lateral sclerosis [5,6]; (b) Rett syndrome [7], a neurodevelopmental disorder that can cause dysfunction of different organs; (c) lung pathologies, such as pneumonia, asthma, and early stages of cancer [5]; and (d) cancers of the gastrointestinal system (esophagus, stomach, colon), as well as Crohn’s disease [5]. The ability of UST to detect organ damage in a wide variety of diseases suggests that it will be generally applicable to detecting post-acute COVID pathologies without limitations to the organs and tissues affected.

**Summary.** Based on our findings, we strongly believe that the UST approach can be useful for prediction, early detection, and monitoring of COVID-19 sequelae. All plasma miRNAs necessary for the analysis of pathology in different organs can be detected in one assay, which makes it not only clinically relevant but also much more efficient than multiple assays for different organs. Further, analysis of epigenetic miRNA biomarkers reflective of underlying pathophysiological processes can lead to better understanding of the pathology.
